# Machine learning to predict postoperative complications after digestive surgery: a scoping review

**DOI:** 10.1093/bjs/znad229

**Published:** 2023-07-21

**Authors:** Maximilien Ravenel, Gaëtan-Romain Joliat, Nicolas Demartines, Emilie Uldry, Emmanuel Melloul, Ismail Labgaa

**Affiliations:** Department of Visceral Surgery, Lausanne University Hospital (CHUV), University of Lausanne (UNIL), Lausanne, Switzerland; Faculty of Biology and Medicine (FBM), University of Lausanne (UNIL), Lausanne, Switzerland; Department of Visceral Surgery, Lausanne University Hospital (CHUV), University of Lausanne (UNIL), Lausanne, Switzerland; Faculty of Biology and Medicine (FBM), University of Lausanne (UNIL), Lausanne, Switzerland; Graduate School of Health Sciences, University of Bern, Bern, Switzerland; Department of Visceral Surgery, Lausanne University Hospital (CHUV), University of Lausanne (UNIL), Lausanne, Switzerland; Faculty of Biology and Medicine (FBM), University of Lausanne (UNIL), Lausanne, Switzerland; Department of Visceral Surgery, Lausanne University Hospital (CHUV), University of Lausanne (UNIL), Lausanne, Switzerland; Faculty of Biology and Medicine (FBM), University of Lausanne (UNIL), Lausanne, Switzerland; Department of Visceral Surgery, Lausanne University Hospital (CHUV), University of Lausanne (UNIL), Lausanne, Switzerland; Faculty of Biology and Medicine (FBM), University of Lausanne (UNIL), Lausanne, Switzerland; Department of Visceral Surgery, Lausanne University Hospital (CHUV), University of Lausanne (UNIL), Lausanne, Switzerland; Faculty of Biology and Medicine (FBM), University of Lausanne (UNIL), Lausanne, Switzerland

## Introduction

Globally, over 13 million individuals undergo digestive surgery each year^1^. Digestive surgery remains associated with a substantial risk of postoperative complications^2^, which has a detrimental impact on costs and on caregivers^3^. Efforts to accurately predict postoperative complications could reduce their impact, and considerable attempts have been made to hone this predictive ability. Unfortunately, results have shown limited performance^4^.

Artificial intelligence (AI) is the broader concept of machines being able to execute tasks intelligently, while machine learning (ML) is a distinct branch of AI that involves training machines to optimize their performance through exposure to data, using algorithms, such as artificial neural networks^5^. Its potent contributions have substantially impacted various fields, including healthcare^6–10^. The aim of the present scoping review was to provide an overview of the available data investigating ML to predict postoperative complications after digestive surgery.

## Methods

This prospectively registered review was conducted in accordance with the current authoritative frameworks for scoping reviews, including studies on the use of ML to predict postoperative complications in digestive surgery. A detailed description of the methods is provided in the *Supplementary Methods*.

## Results

### Study selection

A search of the literature yielded 4327 records. After the application of inclusion and exclusion criteria, a total of 53 articles met the eligibility criteria (*Fig. S1*). Table 1 summarizes these 53 studies.

**Table 1:** Overview of selected study characteristics

	Number of studies	Sample size, median (interquartile range)	Common POC	ML *versus* CS
Upper-GI	5 Bariatric 4 Gastric 1 Oesophagogastric	4334 (919–44 061)	AL (*n* = 5) Overall POC (*n* = 4)	ML > CS in 3 of 4 studies
HPB	8 Pancreatic 5 Liver	552 (159–1322)	POPF (*n* = 8) PHLF (*n* = 3) AKI (*n* = 2)	ML > CS in 5 of 7 studies
Colorectal	20 Colorectal	944 (244–3956)	SSI (*n* = 9) AL (*n* = 6)	ML > CS in 8 of 8 studies
General digestive	6 Emergency surgery 4 Mixed DS	2372 (926–68 224)	Heterogeneous	ML > CS in 4 of 6 studies
Total	53 Studies	1137 (269–5824)	SSI (*n* = 16) AL (*n* = 13)	ML > CS in 20 of 25 studies

POC; postoperative complications; ML, machine learning; CS, conventional statistics; GI, gastrointestinal; AL, anastomotic leakage; HPB, hepatopancreatobiliary; POPF, postoperative pancreatic fistula; PHLF, post-hepatectomy liver failure, AKI, acute kidney injury; SSI, surgical site infection; DS, digestive surgery.

### Characteristics of sources of evidence

**Fig. 1:**
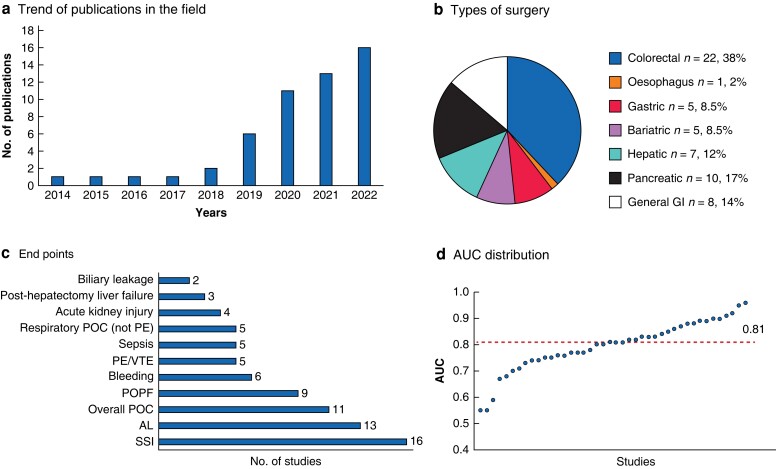
Characteristics of the selected studies **a** Number of articles published per year on the topic. **b** Distribution of the selected studies according to the types of digestive surgery. **c** Selected endpoints and their frequency. **d** Distribution of the values of the area under the curve for the reported machine-learning models that aimed to predict postoperative complications after digestive surgery. GI, gastrointestinal; POC, postoperative complications; PE, pulmonary embolism; VTE, venous thromboembolism; POPF, postoperative pancreatic fistula; AL, anastomotic leakage; SSI, surgical site infection; AUC, area under the curve.

The topic has gained major interest over the last years, with most studies (47 of 53, 87 per cent) being published from 2019 onwards (*Fig. 1a*). The distribution of these studies based on the type of surgery is detailed in *Fig. 1b*. The most frequently investigated endpoints are illustrated in *Fig. 1c*. Sample sizes were heterogeneous, ranging from 32^11^ to 1 003 703 patients^12^. Various ML algorithms were established, including artificial neural networks (24, 45 per cent), gradient-boosted machines (24, 45 per cent), and random forests (22, 42 per cent). The area under the curve (AUC) of the model was provided by 44 studies (83 per cent), showing a median value of 0.81 (0.75–0.87) (*Fig. 1d*), and compared with conventional statistical methods in 25 (47 per cent) studies.

### Upper-gastrointestinal surgery

A total of 10 articles were identified, with 5 studies involving bariatric surgery, 4 studies involving gastric surgery and 1 study involving oesophagogastric surgery (*Table S2*). Anastomotic leakage (AL) is an important issue after upper-gastrointestinal surgery, associated with substantial consequences. Integrating demographics, medical history, laboratory tests, and surgical details, Shao *et al*.^13^ established an ML model to predict AL after gastrectomy, showing a good performance, with an AUC of 0.90. In a large cohort of patients undergoing bariatric surgery, ML showed a better predictive value for AL compared with a linear model (AUC 0.75 *versus* 0.63 respectively, *P* < 0.001)^14^. Nudel *et al*.^14^ utilized a nationwide database from the USA to analyse the predictive value of ML for AL and venous thromboembolism (VTE) after bariatric surgery. For both types of complications, ML outperformed linear models (AUC = 0.75 *versus* 0.63 respectively for AL and AUC = 0.67 *versus* 0.64 respectively for VTE).

### Hepatopancreatobiliary surgery

A total of 13 studies were included in this section (*Table S3*). Preoperative imaging is often available for patients undergoing pancreatic surgery and this type of data can be used to predict surgical outcomes. Among the studies that applied imaging to postoperative pancreatic fistula (POPF) prediction algorithms, three groups of researchers integrated imaging data in ML algorithms and showed promising predictive values. As an example, a proof-of-concept of this approach was investigated in a pilot study of 110 patients undergoing pancreatoduodenectomy, equally matched for POPF (55 patients with POPF and 55 patients without POPF)^15^. The imaging-based model showed excellent performance, with an AUC of 0.95, with a sensitivity and specificity of 96 and 98 per cent respectively.

A total of five studies (38 per cent) were conducted in patients undergoing liver surgery; three of these studies (60 per cent) explored the application of ML models to tackle the challenging complication of post-hepatectomy liver failure (PHLF). In a cohort of 353 patients with hepatocellular carcinoma (‘HCC’), ML showed a valuable performance, with an AUC of 0.88 compared with 0.79 (*P* < 0.050) in a linear model^16^.

### Colorectal surgery

A total of 20 studies were included in this section (*Table S4*). The Mayo Clinic group used the American College of Surgeons’ National Surgical Quality Improvement Program (ACS-NSQIP) to build an ML model that aimed to predict surgical site infection after colorectal surgery^17^. They reported a good performance, with an AUC of 0.83, which outperformed a linear model (AUC = 0.72). Other studies leveraged ML to predict AL, showing promising results. As an illustration, an algorithm developed in a cohort of 5220 patients undergoing anterior resection for rectal cancer showed an AUC of 0.87 to predict postoperative AL, as opposed to an AUC of 0.72 for linear regression^18^.

### General digestive surgery

This section included 10 studies (*Table S5*). A total of four of six (66 per cent) studies comparing ML with linear models highlighted the higher performance of the ML algorithms.

In a large-scale study analysing 246 124 patients from the NSQIP database (197 488 patients for colectomy, 25 403 for hepatectomy, and 23 333 for PD), AL advantageously predicted biliary leakage (AUC = 0.75 *versus* 0.72, *P* < 0.001), POPF (AUC = 0.75 *versus* 0.71, *P* = 0.003), and AL (AUC = 0.68 *versus* 0.63, *P* = 0.001) compared with linear regression^19^.

The ML-based Predictive OpTimal Trees in Emergency surgery Risk (‘POTTER’) calculator is an externally validated risk-assessment tool, which was also developed from the ACS-NSQIP database, which showed promising performance in predicting mortality and morbidity^20^.

## Discussion

The use of ML to predict postoperative complications after digestive surgery found 53 studies that demonstrated a feasible and promising approach. Moreover, ML appeared as a polyvalent tool capable of predicting different types of postoperative complications in various settings.

Despite growing interest and a rise in publications over the past 5 years, data on the subject remain scant. Furthermore, the included studies showed significant heterogeneity. While ML may offer superior performance, its success hinges on the quality of input data. Thus identifying new potent biomarkers is paramount for improving the prediction of postoperative complications, a challenge that ML alone cannot solve. Also, ML offers unique opportunities to exploit new sources of input data for the prediction of postoperative complications, such as intraoperative video samples. The heterogeneous designs translated into heterogeneous performances of the models, with a wide range of AUC values. Nonetheless, the median AUC reached the encouraging value of 0.81, and ML showed a higher performance than linear models in the majority of available comparisons (20 of 25).

Future efforts in the field must focus on conducting studies including independent cohort for external validation. Particularly, the clinical impact and standard requirements for performance, such as AUC values, must be investigated and determined. Also, examining ML performance across subgroups, namely oncological/non-oncological and elective/emergent patients, could expose the need for distinct algorithms adapted to specific clinical scenarios. Many included studies lacked rigorous and transparent descriptions of ML algorithm development and data preparation methods. This is critical because the same algorithms can produce different outcomes based on their implementation, emphasizing the need for in-depth understanding to progress in future research.

## Supplementary Material

znad229_Supplementary_Data

## Data Availability

Data used in this article are already publicly available.
