# Supplementary material for: Machine learning to predict postoperative complications after digestive surgery: a scoping review
Source: Br J Surg. 2023 Jul 21;110(12):1646–9. doi: 10.1093/bjs/znad229 (PMC10638531; doi:10.1093/bjs/znad229)
Supplement: znad229_Supplementary_Data [file znad229_supplementary_data.docx]

**Title: Machine Learning to Predict Postoperative Complications After Digestive Surgery: A Scoping Review**

Authors**:** Maximilien Ravenel, M.S. ^1, 2^, Gaëtan-Romain Joliat, M.D. ^1, 2, 3^, Nicolas Demartines, M.D. ^1, 2^, Emilie Uldry, M.D. ^1, 2^, Emmanuel Melloul, M.D. ^1, 2^, Ismail Labgaa, M.D., Ph.D. ^1, 2^

^1^ Department of Visceral Surgery, Lausanne University Hospital (CHUV), University of Lausanne (UNIL), Switzerland.

^2^ Faculty of Biology and Medicine (FBM), University of Lausanne (UNIL), Lausanne, Switzerland

^3^ Graduate School of Health Sciences, University of Bern, Switzerland.

**Corresponding author.**

Ismail Labgaa, MD, PhD

Department of Visceral Surgery

Lausanne University Hospital (CHUV)

Rue du Bugnon 46

CH-1011 Lausanne

P: +41 (0)21 314 24 28

M: +41 (0)79 556 68 47

ORCID ID: 0000-0003-4286-2170

Twitter: @LabgaaI

**Supplementary Materials - Index**

| **Supplementary Methods** |  |
| --- | --- |
| Protocol and registration | *page 3* |
| Eligibility criteria | *page 3* |
| Information sources and search  Study selection  Data Charting process and items  Synthesis of results  Search algorithms | *page 3*  *page 3*  *page 4*  *page 4*  *page 4* |
|  |  |
|  |  |
|  |  |
|  |  |
| **Supplementary Figures and Tables** |  |
| Table S1 | *page 5* |
| Table S2  Table S3  Table S4 | *page 8*  *page 11*  *page 15* |
| Table S5 | *page 20* |
| Figure S1 | *page 23* |
| **References** | *page 24* |
|  |  |
|  |  |
|  |  |
|  |  |
|  |  |
|  |  |
|  |  |

**Supplementary Methods**

**Protocol and registration**

This scoping review was conducted and reported in accordance with the Arksey and O’Malley’s framework for scoping reviews ^1^, the subsequent amendments made by the Joanna Briggs Institute ^2^, and PRISMA-ScR (Preferred Reporting Items for Systematic Reviews and Meta-Analyses extension for Scoping Reviews) ^3^. The Protocol was prospectively registered on the Open Science Framework (OSF) on December 31, 2022: <https://doi.org/10.17605/OSF.IO/7XQKN>. A PDF of the protocol is available under *files – archive of OFS storage*.

**Eligibility criteria**

Main inclusion criterion was the use of ML to predict POC in patients undergoing DS. ML was defined according to a predefined list of algorithms qualified as ML and their related algorithms referred to in Table S1. DS was defined as upper-gastrointestinal (upper-GI: esophagus, gastric or bariatric), hepato-pancreatico-biliary (HPB) or colorectal surgery. Studies in emergency general surgery or abdominal trauma patients were also included. Studies providing non-human data (i.e. *in vitro* and *in vivo* studies) were excluded. Only original peer-reviewed data were considered whereas reviews, commentaries, editorials and meta-analyses were excluded. Studies in critical care, transplantation or lacking full-text manuscript were also excluded.

**Information sources and search**

Search was run in the following databases: MEDLINE, EMBASE, Google Scholar and Web of Science, until January 21, 2023. Only the first 400 results from the Google scholar database were scanned, as previously described ^4^. Cross-referencing was also performed.

**Study selection**

Study selection was independently performed by 2 investigators (MR and GRJ). In case of disagreement, a consensual decision was taken with a third co-investigator (IL). Potentially relevant sources from MEDLINE, EMBASE, Google Scholar and Web of Science were retrieved and imported into Zotero (6.0.18) where duplicates were removed. Full texts of the studies meeting inclusion criteria were imported for a thorough review.

**Data charting process and items**

Data were extracted by MR and validated by IL. All items were predetermined, as in the protocol published on OSF. In the present article, the following variables were provided in the tables: (I) – Authors, (II) – Type of surgery, (III) Number of included patients (Training cohort – Validation cohort – Testing cohort), (IV) – Types of data integrated in the models and (V) – Main findings. Following guidelines for scoping reviews, methodological quality of the selected studies was not assessed ^1,3^.

**Synthesis of results**

Results were classified according to the type of surgery and provided in a narrative form. Details of each selected studies are also provided in **Tables S2-5**. Descriptive statistics were performed. Continuous variables were provided either as median [interquartile range] or mean [standard deviation] values, according to their distribution. External validation studies were not included in these numbers. Categorical variables were provided as frequencies with percentages. All statistics were performed with IBM SPSS Statistics, version 27.0.

**Search algorithms**

Medline algorithm:

*("Artificial Intelligence"[Mesh] OR "Machine Learn*"[tiab] OR "Neural Network*"[tiab] OR "Artificial Intelligence"[tiab]) AND ("Postoperative Complications"[Mesh] OR "postoperative complication*"[tiab] OR "post-operative complication*"[tiab])*

EMBASE algorithm:

*("Artificial Intelligence"/exp OR "Machine Learn*":ab,ti OR "Neural Network*":ab,ti OR "Artificial Intelligence":ab,ti) AND ("Postoperative Complications"/exp OR "postoperative complication*":ab,ti OR "post-operative complication*":ab,ti)*

Web of Science algorithm:

*((TS=(artificial intelligence) OR TS=(machine learning) OR TS=(Neural networks)) AND (TS=(post operative complication)))*

Google Scholar algorithm:

*"machine learning" OR "artificial intelligence" "postoperative complication"*

**Supplementary Figures and Tables**

**Supplementary Table 1: Included machine learning algorithms**

| ANN = Artificial Neural Network | An umbrella term for neural networks. Similarly to a biological brain, neurons are interconnected with dendrites and axons and organised in layers. ANNs consist of an input layer, hidden layers and an output layer. Each neuron contains scalable weights which will contribute to the output decision. This process can be repeated to find the most accurate model. Examples include MLP, RNN, CNN^58^. |
| --- | --- |
| DT = Decision Trees | A flowchart-like model that from a root node, splits off into internal nodes classifying data into progressively smaller subsets until it reaches terminal nodes called leaves which represent a decision. It can be used for both classification and regression tasks and is favoured for its high interpretability^59^. |
| GBM = Gradient Boosting Machine | An umbrella term for a subtype of boosting algorithms. GBM typically use DTs, but also encompass linear models and produces simple algorithms sequentially in which every new one learns from the errors of the last. Gradient refers to the method used to calculate predictive error on which to base new parameters for the next algorithm. Boosting refers to the method of sequential learning and averaging of the results of all the algorithms. Examples include XGB, LightGBM, CatBoost^60^. |
| RF = Random Forest | A model that is based on the parallel production of a great number of DTs with specific predictors. The final decision is made by averaging the results of the individual trees. The averaging in addition to the parallel nature of the DT production is called bagging^61^. |
| SVM = Support Vector Machine | A model that used for classification and regression analysis. It uses a hyperplane with maximal margins to distinguish two groups of datapoints and to make decisions with new unlabelled data^62^. |
| K-NN = K-Nearest Neighbour | A model mostly used for classification, although it can also be used for regression analysis. It stores a training dataset and classifies new data based on a majority vote i.e. based on the label most represented near the testing datapoint. "K" is the number of datapoints closest to the testing data used for evaluation^63^. |
| NB = Naïve Bayes | A family of classification models based on "Bayes’ theorem" of probabilities. Naïve comes from the model's assumption that all features are independent from each other^64^. |

Table 1: Definition of most frequently used machine learning algorithms in the prediction of postoperative complications. CNN: Convolultional Neural Network, LightGBM: Light Gradient Boosting Machine, MLP: MultiLayer Perceptron, RNN: Recursive Neural Network, XGB: eXtreme Gradient Boosting.

**Supplementary Table 2: Upper-GI surgery (n=10 studies)**

| Reference | Type of surgery | Number of patients | Types of data integrated in the models | Main findings |
| --- | --- | --- | --- | --- |
| Celik and al. ^6^ | Gastric | TC: 108  VC: NA  TeC: 90  **Total: 198** | - Demographics - Clinical data - Surgical details | - The ML Naïve Bayes and Random Forest models outperformed other ML models in predicting **AL**. |
| Lu and al. ^7^ | Gastric | TC: 108  VC: NA  TeC: 90  **Total: 321** | - Demographics - Medical history - Clinical data - Laboratory values - Surgical details | - ML outperformed linear models in predicting **POC** (Clavien-Dindo grade ≥ II) with AUC=0.80 *vs.* 0.71. |
| Fukuyo and al. ^8^ | Gastric | TC: 509  VC: NA  TeC: 218  **Total: 727** | - Demographics - Medical history - Clinical data - Laboratory values - Surgical details - Pathology | - ML predicted **major** **POC** with AUC=0.71.It also predicted **major intra-abdominal** **POC** with AUC=0.77. |
| Shao and al. ^9^ | Gastric | TC: 1'328  VC: NA  TeC: 332  **Total: 2’240** | - Demographics - Medical history - Clinical data - Laboratory values - Surgical details | - The best ML model predicted **AL** with AUC=0.90. - All other models showed AUC >0.80. |
| van Kooten and al. ^5^ | Oesogastric | TC: 4'820  VC: NA  TeC: 1'609  **Total: 6’427** | - Demographics - Medical history - Clinical data - Surgical details - Pathology | - Linear models slightly outperformed ML in predicting **AL**. - Gastric: AUC=0.680 *vs.* 0.678 - Oesophageal: AUC=0.619 *vs.* 0.617 - The linear model also outperformed ML in predicting **pulmonary complications** post oesophageal surgery (AUC=0.644 *vs.* 0.639. |
| Sheikh-taheri and al. ^10^ | Bariatric  (GBP) | TC: 1'045  VC: 224  TeC: 224  **Total: 1’493** | - Demographics - Medical history - Clinical data - Laboratory values - Surgical details - Endoscopy data | - ML models for the prediction of **POC** at 10 days showed accuracy, sensitivity and specificity of 98.4%, 98.6%, and 98.3%, respectively |
| Cao and al. ^11^ | Bariatric | TC: 37'811  VC: NA  TeC: 6'250  **Total: 44’061** | - Demographics - Medical history - Laboratory values - Surgical details | - Of the 29 models tested, ensemble (combined) models tended to work best in predicting **severe POC**. - The Deep Neural Network model held the most promise among all models to improve in predictive accuracy. |
| Cao and al. ^12^ | Bariatric | TC: 37'811  VC: 5-fold CV  TeC: 6'250  **Total: 44’061** | - Demographics - Medical history - Laboratory values - Surgical details | - The best Neural Network model predicted **serious POC** (grade ≥ IIIb) post bariatric surgery with AUC 0.55. |
| Wise and al. ^13^ | Bariatric  (SG) | TC: 81'376  VC: K-fold CV  TeC: 20'344  **Total: 101’721** | - Demographics - Medical history | - ML slightly improved prediction of POC after sleeve gastrectomy, compared to linear model (AUC=0.59, 0.57) |
| Nudel and al. ^14^ | Bariatric | TC: 218’403  VC: 109’202  TeC: 109’202  **Total: 436’807** | - Demographics - Medical history - Laboratory values - Surgical details | - The best ML model outperformed the linear model (AUC=0.75 *vs.* 0.63) p<0.001) in predicting **AL** after bariatric surgery. - ML also slightly outperformed the linear model in predicting postoperative **VTE** (AUC=0.67 *vs.* 0.64, p<0.001). |

Table 2: Abbreviations: AL: Anastomotic Leak, AUC: Area Under the Curve, CV: Cross-Validation, ML: Machine Learning, NA: Not Available, OP: Operative, POC: PostOperative Complication, POD: PostOperative Day, TC: Training Cohort, TeC: Testing Cohort, VC: Validation Cohort, VTE: Venous ThromboEmbolism.

**Supplementary Table 3: HPB surgery (n=13 studies)**

| Reference | Type of surgery | Number of patients | Types of data integrated in the models | Main findings |
| --- | --- | --- | --- | --- |
| Skawran and al. (32) | Pancreas  (DP) | TC: 126*  VC: NA  TeC: 54*  **Total: 62** | - Imaging | - Combining radiomics and T1 SiRatio in a ML model predicted CR-POPF with AUC=0.90. |
| Capretti and al. ^15^ | Pancreas  (DP) | TC: 100  VC: NA  TeC: 10-fold **CV Total: 100** | - Demographics - Imaging - Surgical details | - The linear model outperformed the ML model in predicting **CR-POPF**, with AUC=0.81 *vs.* 0.75. |
| Kamba-kamba and al. ^16^ | Pancreas  (DP) | TC: 110  VC: NA  TeC: 10-fold CV  **Total: 110** | - Imaging | - Texture analysis via ML techniques outperformed clinical fistula risk scores with excellent performance in predicting **POPF**, with AUC=0.95 *vs.* 0.80. |
| Pfitzner and al. ^17^ | Pancreas | TC: 521  VC: NA  TeC: LOO CV  **Total: 521** | - Demographics - Medical history - Laboratory values - Vitals monitoring - Surgical details | - Using only preoperative and intraoperative data, ML outperformed the linear model in predicting **POPF**, but not PPH. - By adding postoperative and dynamic data, ML outperformed the linear models in predicting both **POPF** and **PPH**. |
| Mu and al. ^18^ | Pancreas  (DP) | TC: 359  VC: 154  TeC: 70**  **Total: 583** | - Imaging | - ML model outperformed the fistula risk score in predicting **CR-POPF** (AUC=0.89 *vs.* 0.73, p<0.001). |
| Han and al. ^19^ | Pancreas  (DP) | TC: 1'769  VC: NA  TeC: 5-fold CV  **Total: 1’769** | - Demographics - Medical history - Laboratory values - Surgical details - Imaging results | - The best ML model predicted **POPF** with AUC=0.74. |
| Shen and al. ^20^ | Pancreas  (DP) | TC: 2'421  VC: NA  TeC: 5-fold CV  **Total: 2’421** | - Demographics - Medical history - Clinical data - Laboratory values - Surgical details | - ML model based on pre- and intra-operative data predicted POPF with AUC=0.68. - Adding postoperative factors to the model increased AUC to 0.83. |
| Yoon** and al. ^21^ | Pancreas  (DP) | TC: NA  VC: NA  TeC: 1'576***  **Total: 1'576** | - Demographics - Medical history - Laboratory values - Surgical details - Imaging results | - External validation of a previously published algorithm for the prediction of POPF (Han et al. (35)). - This validation study reported an AUC=0.67 *vs.* 0.74, in the training study. |
| Zeng and al. ^22^ | Liver | TC: 122  VC: NA  TeC: 53  **Total: 175** | - Demographics - Laboratory values - Surgical details - Pathology | - The best ML model predicted **POC** with AUC=0.91, in patients with liver cancer. |
| Mai and al. ^23^ | Liver | TC: 265  VC: NA  TeC: 88  **Total: 353** | - Laboratory values - Standardised future liver remnant | ML outperformed linear model in predicting **severe PHLF** in HCC patients, with AUC=0.88 *vs.* 0.79, p<0.05.   - It also outperformed commonly used tools such as the Child-Pugh, MELD, ALBI, FIB-4 and APRI scores. |
| Wang and al. ^24^ | Liver | TC: 612  VC: 88  TeC: 175  **Total: 875** | - Demographics - Medical history - Clinical data - Laboratory values - Surgical details - PHLF score | - Machine learning predicted **PHLF** with AUC=0.82. |
| Lei and al. ^25^ | Liver | TC: 821  VC: NA  TeC: 352  **Total: 1’173** | - Demographics - Medical history - Laboratory values - Surgical details | - The best ML model predicted **AKI** after HCC resection with AUC=0.77, in patients with liver cancer. |
| Dong and al. ^26^ | Liver | TC: 1'715  VC: 10-fold CV  TeC: 735  **Total: 2’450** | - Demographics - Medical history - Clinical data - Laboratory values - Surgical details | - The best ML model outperformed the linear model in predicting AKI after HCC resection, AUC=0.92 *vs.* 0.85. |

Table 3: *Augmented data. ** External validation cohort. ***Validation study, the index study is Han^19^. Abbreviations: AKI: Acute Kidney Injury, ALBI: Albumin-Bilirubin, APRI: Aspartate transaminase to Platelet Ratio Index, AUC: Area Under the Curve, CR-POPF: Clinicaly Relevant Postoperative Pancreatic Fistula, CV: Cross-Validation, FIB-4: Fibrosis 4, LOO: Leave-One-Out, MELD: Model for End-stage Liver Disease, ML: Machine Learning, NA: Not Available, PD: PancreaticoDuodenectomy, PHLF: PostHepatectomy Liver Failure, POC: PostOperative Complication, POPF: PostOperative Pancreatic Fistula, POD: PostOperative Day, PPH: PostPancreatectomy Haemorrhage, TC: Training Cohort, TeC: Testing Cohort, VC: Validation Cohort.

**Supplementary Table 4: Colorectal surgery (n=20 studies)**

| Reference | Type of surgery | Number of patients | Types of data integrated in the models | Main findings |
| --- | --- | --- | --- | --- |
| Sofo and al. ^27^ | Colorectal  (Total colectomy for UC) | TC: 32  VC: NA  TeC: LOO CV  **Total: 32** | - Demographics - Clincal data - Laboratory values - Surgical details - Body temperature | - ML model based on preoperative data showed sensitivity of 87.5% and specificity of 83.3% for the prediction of **minor POC,** in patients. |
| Mizuno and al. ^28^ | Colorectal  (IPAA for UC) | TC: 43  VC: NA  TeC: 5-fold CV  **Total: 43** | - Demographics - Clinical data - Surgical details - Endoscopy data | - ML outperformed mPDAI for the prediction of pouchitis (AUC=0.84 vs. 0.62). |
| Hosaka and al. ^29^ | Colorectal | TC: 51  VC: NA  TeC: NA  **Total: 51** | - Demographics - Medical history - Clinical data - Laboratory values - Surgical details | - ML model mainly based on lactates and albuminemia showed an AUC=0.83, to predict **POC** in patients undergoing emergency surgery for digestive perforation. |
| Adams and al. ^30^ | Colorectal | TC: 76  VC: NA  TeC: 12  **Total: 76** | - Demographics - Surgical details - Laboratory values - Vitals monitoring | - A pilot study showing the applicability of ML to predict clinical AL (AUC=0.89). |
| Azimi and al. ^31^ | Colorectal | TC: 166  VC: NA  TeC: 42  **Total: 208** | - Demographics - Medical history - Clinical data - Laboratory values - Surgical details | - ML based on preoperative data showed higher performance compared to linear model for the prediction of SSI with AUC=0.81 *vs.* 0.79. |
| Mazaki and al. ^32^ | Colorectal | TC: 156  VC: NA  TeC: 100  **Total: 256** | - Demographics - Laboratory values - Surgical details - Pathology | - The ML model predicted **AL** with an AUC of 0.77. |
| Patrascu and al. ^33^ | Colorectal | TC: 225  VC: 28  TeC: 28  **Total: 281** | - Demographics - Clinical data - Laboratory values - Pathology | - Prediction of **AL**, **POC** and **POC severity** was enhanced by integrating ML into the algorithm with an accuracy of 0.79, 0.71 and 0.70, respectively. |
| Soguero-Ruiz and al. ^34^ | Colorectal | TC: 61  VC: LOO CV  TeC: 340  **Total: 402** | - Free text - Laboratory values - Vitals monitoring | - The ML model predicted **AL** with AUC=0.83, on POD 0. - Adding postoperative data increased AUC to 0.88, on POD 4. |
| Ohno and al. ^35^ | Colorectal | TC: 730  VC: NA  TeC: 100  **Total: 730** | - Demographics - Medical history - Clinical data - Laboratory values - Surgical details - Pathology | - The ML model predicted **SSI** with AUC=0.73, after colon cancer resection. |
| Sohn and al. ^36^ | Colorectal | TC: 751  VC: NA  TeC: 10-fold CV  **Total: 751** | - Demographics - Medical history - Surgical details | - ML outperformed the linear model in predicting **SSI**, with AUC=0.83 *vs.* 0.72. - ML showed better performance for **clinically relevant SSI** (AUC=0.89). |
| Kocbek and al. ^37^ | Colorectal | TC: 1'137  VC: NA  TeC: 5-fold CV  **Total: 1’137** | - Laboratory values | - All models showed excellent performance in predicting **SSI**, with mean AUC=0.956. |
| Dudurych and al. ^38^ | Colorectal  (Pelvic exenteration) | TC: 688  VC: 229  TeC: 230  **Total: 1’147** | - Demographics - Clinical data - Surgical details - Pathology | - ML slightly outperformed the linear model in predicting **30-day POC** rates with AUC=0.76 *vs.* 0.74. |
| Shao and al. ^39^ | Colorectal  (AR) | TC : 1’145  VC : NA  TeC : 550/545  **Total : 2'240** | - Demographics - Medical history - Clinical data - Laboratory values - Surgical details | - ML algorithm to guide surgeons in the decision-making of ileostomy, tested in two cohorts with AUC of 0.78 and 0.88. |
| Grass and al. ^40^ | Colorectal | TC: 2'376  VC: NA  TeC: 10-fold CV  **Total: 2’376** | - Demographics - Medical history - Clinical data - Laboratory values - Surgical details | - ML trained on institutional data outperformed the linear model in predicting **deep SSI**, with AUC=0.78 *vs.* 0.71. - In the external dataset, AUC decreased to 0.74. |
| Ruan and al. ^41^ | Colorectal | TC: 3'234  VC: NA  TeC: 300  **Total: 3’534** | - Demographics - Medical history - Laboratory values - Vitals monitoring - Surgical details | - ML outperformed the linear model in predicting **organ space SSI** and **wound infection**. - The linear model outperformed ML in predicting **postoperative bleeding** and **superficial SSI**. |
| Wen and al. ^42^ | Colorectal  (AR) | TC: 4'384  VC: 5-fold CV  TeC: 836  **Total: 5’220** | - Demographics - Medical history - Clinical data - Laboratory values - Surgical details | - ML outperformed nomogram constructed from the same data in predicting **AL**, with AUC 0.87 *vs.* 0.72. |
| Weller and al. ^43^ | Colorectal | TC: 7'748  VC: NA  TeC: 1'850  **Total: 9’598** | - Demographics - Medical history - Clinical data - Laboratory values - Surgical details | - ML predicted several adverse events, with AUC=0.86 for **bleeding**, AUC=0.71 for **ileus** and AUC=0.56 for **SSI**. |
| Chen and al. ^44^ | Colorectal | TC: 13'399  VC: NA  TeC: 10-fold CV  **Total: 13’399** | - Demographics - Medical history - Clinical data - Laboratory values - Vitals monitoring - Surgical details | - ML outperformed the linear model in predicting **postoperative bleeding,** with AUC 0.82 *vs.* 0.77, p<0.001. |
| Lin and al. ^45^ | Colorectal | TC: 17'930  VC: 3-fold CV  TeC: 5'977  **Total: 23’907** | - Demographics - Medical history - Clinical data - Surgical details - Pathology | - ML predicted **severe POC** (grade > IIIb) with AUC=0.70 and **AL** with AUC=0.69.   . |
| Chen and al. ^46^ | Colorectal | TC: 220'122  VC: 5-fold CV  TeC: 55'030  **Total: 275’152** | - Demographics - Medical history - Clinical data - Laboratory values - Surgical details | - ML outperformed the linear model in predicting **SSI**, with AUC 0.77 *vs.* 0.68, p<0.001. |

Table 4: *12 patients were used for external validation. Abbreviations: AL: Anastomotic Leak, AR: Anterior Resection, AUC: Area Under the Curve, CV: Cross-Validation, IPAA: Ileal Pouch-Anal Anastomosis, LOO: Leave-One-Out, NA: Not Available, POC: PostOperative Complication, POD: PostOperative Day, SSI: Surgical Site Infection, TC: Training Cohort, TeC: Testing Cohort, UC: Ulcerative Colitis, VC: Validation Cohort.

**Supplementary Table 5: General digestive surgery (n=10 studies)**

| Reference | Type of surgery | Number of patients | Types of data integrated in the models | Main findings |
| --- | --- | --- | --- | --- |
| Gelbard and al. ^47^ | Laparotomy for abdominal trauma | TC: 132  VC: NA  TeC: LOO CV  **Total: 132** | - Laboratory values - PATI score | - ML outperformed linear model in predicting **severe sepsis** (AUC=0.80 *vs.* 0.75), and **organ space infection** (AUC=0.77 *vs.* 0.64). |
| Eickhoff and al. ^48^ | Perforated appendicitis | TC: 163  VC: NA  TeC: 10-fold CV  **Total: 163** | - Demographics - Medical history - Clinical data - Laboratory values - Surgical details | - ML model was associated with an accuracy of 68% for **major POC**, 66% for **SSI** and 77%, for **the need of ICU**. |
| Xue and al. ^49^ | Emergency surgery | TC: 648  VC: 5-fold CV  TeC: 278  **Total: 926** | - Demographics - Clinical data - Laboratory values - Surgical details | - ML and the linear model showed identical performance for the prediction of **respiratory POC** (AUC=0.81). |
| Soguero-Ruiz and al. ^50^ | GI | TC: 101 x 5  VC: LOO CV  TeC: 25 x 5  **Total: 1’005** | - Laboratory values | - Non-linear Support Vector Machine (ML) was identified as the best model to predict **SSI** (pre-operative accuracy = 0.87). |
| Deng and al. ^51^ | Cytoreductive surgery | TC: 1'897  VC: NA  TeC: 479  **Total: 2’372** | - Demographics - Medical history - Clinical data - Laboratory values - Surgical details | - ML slightly outperformed linear model in predicting **severe POC**   (AUC 0.74 *vs.* 0.71). |
| Merath and al. ^52^ | Colorectal, hepato-pancreatic | TC: 15'657  VC: NA  TeC: NA  **Total: 15’657** | - Demographics - Medical history - Clinical data - Laboratory values - Surgical details | - ML outperformed ACS-SRC in predicting **POC** (c-statistic 0.74 *vs.* 0.71). |
| Chen and al. ^53^ | Colorectal, hepato-pancreatic | TC: 147'675  VC: 49'224  TeC: 49'225  **Total: 246’124** | - Demographics - Medical history - Clinical data - Laboratory values - Surgical details | - ML outperformed linear model in predicting: - **Bile leakage**: AUC 0.75 vs 0.72, p<0.001 - **POPF**: AUC 0.75 vs 0.71, p=0.003 - **AL**: AUC 0.68 vs 0.63, p=0.001 |
| Bunn and al. ^54^ | Appendectomy | TC: 178'571  VC: NA  TeC: 44'643  **Total: 223’214** | - Demographics - Medical history - Clinical data - Laboratory values | - ML and the linear model showed equally predicted sepsis (AUC=0.70). |
| Hadaya and al. ^55^ | Emergency surgery | TC: 752'777  VC: NA  TeC: 250'926  **Total: 1’003’703** | - Demographics - Medical history - Social data - Surgical details | - ML slightly outperformed the linear model to predict **acute respiratory failure** after emergency general surgery (AUC 0.90 *vs.* 0.89). |
| El Hechi* and al. ^56^ | Emergency surgery | TC: NA  VC: NA  TeC: 59'955  **Total: 59’955** | - Demographics - Medical history - Clinical data - Laboratory values - Surgery data | - Validation of the POTTER tool in emergency surgery, showing the following c-statistic values: - Mortality (30 day)=0.93 - Morbidity=0.83 - Septic shock=0.93 - Acute renal failure=0.92 |

Table 5: *Validation study, index study by Bertsimas ^57^. Abbreviations: ACS-SRC: American College of Surgeons Surgical Risk Calculator, AL: Anastomotic Leak, ASA: American Society of Anaesthesiologists, AUC: Area Under the Curve, CV: Cross-Validation, GI: GastroIntestinal, ICU: Intensive Care Unit, LOO: Leave-One-Out, ML: Machine Learning, NA: Not Available, PATI score: Penetrating Abdominal Trauma Index score, POC: PostOperative Complication, POPF: PostOperative Pancreatic Fistula, POTTER: Predictive OpTimisation Tree in Emergency surgery Risk, SSI: Surgical Site Infection, TC: Training Cohort, TeC: Testing Cohort, VC: Validation Cohort.


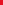


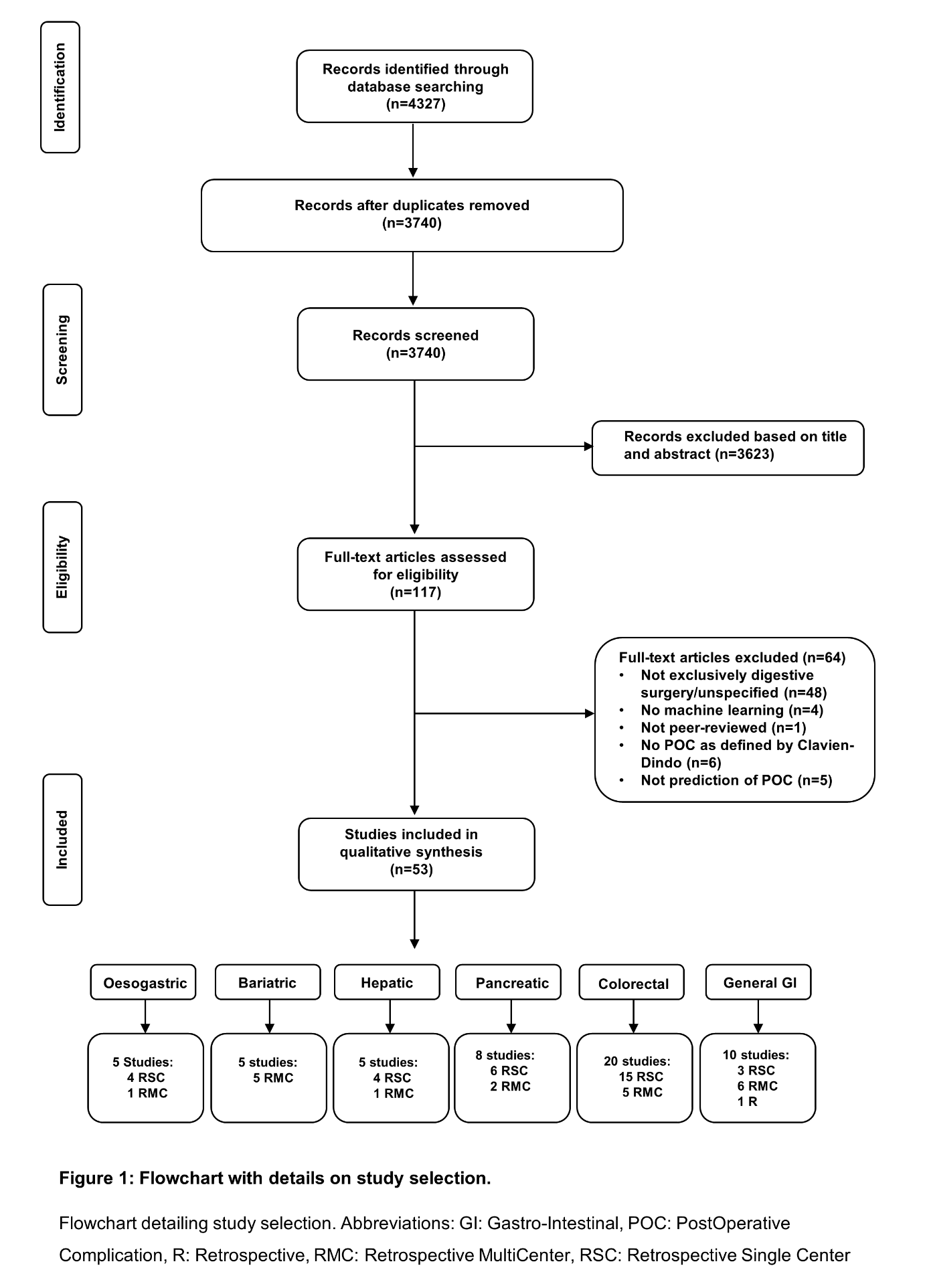


**References**

1. Arksey H, O’Malley L. Scoping studies: towards a methodological framework. International Journal of Social Research Methodology. 2005 Feb;8(1):19–32.

2. Peters MDJ, Godfrey C, McInerney P, Munn Z, Tricco AC, Khalil, H. Chapter 11: Scoping reviews (2020 version). In: JBI Manual for Evidence Synthesis. JBI; 2020.

3. Tricco AC, Lillie E, Zarin W, O’Brien KK, Colquhoun H, Levac D, et al. PRISMA Extension for Scoping Reviews (PRISMA-ScR): Checklist and Explanation. Ann Intern Med. 2018 Oct 2;169(7):467–473.

4. Muka T, Glisic M, Milic J, Verhoog S, Bohlius J, Bramer W, et al. A 24-step guide on how to design, conduct, and successfully publish a systematic review and meta-analysis in medical research. Eur J Epidemiol. 2020 Jan;35(1):49–60.

5. van Kooten RT, Bahadoer RR, Ter Buurkes de Vries B, Wouters MWJM, Tollenaar RAEM, Hartgrink HH, et al. Conventional regression analysis and machine learning in prediction of anastomotic leakage and pulmonary complications after esophagogastric cancer surgery. J Surg Oncol. 2022 Sep;126(3):490–501.

6. Celik S, Sohail A, Ashraf S, Arshad A. Application of machine learning techniques to analyze anastomosis integrity after Total gastrectomy for prediction of clinical leakage. Health Technol. 2019;9(5):757–763.

7. Lu S, Yan M, Li C, Yan C, Zhu Z, Lu W. Machine-learning-assisted prediction of surgical outcomes in patients undergoing gastrectomy. Chin J Cancer Res. 2019 Oct;31(5):797–805.

8. Fukuyo R, Tokunaga M, Umebayashi Y, Saito T, Okuno K, Sato Y, et al. Deep learning-based diagnostic model for predicting complications after gastrectomy. Asian J Endosc Surg. :2023 Apr;16(2):210-217.

9. Shao S, Liu L, Zhao Y, Mu L, Lu Q, Qin J. Application of Machine Learning for Predicting Anastomotic Leakage in Patients with Gastric Adenocarcinoma Who Received Total or Proximal Gastrectomy. JPM. 2021 Jul 29;11(8):748.

10. Sheikhtaheri A, Orooji A, Pazouki A, Beitollahi M. A Clinical Decision Support System for Predicting the Early Complications of One-Anastomosis Gastric Bypass Surgery. Deep learning-based diagnostic model for predicting complications after gastrectomy. :2019 Jul;29(7):2276-2286.

11. Cao Y, Fang X, Ottosson J, Näslund E, Stenberg E. A Comparative Study of Machine Learning Algorithms in Predicting Severe Complications after Bariatric Surgery. J Clin Med. 2019 May 12;8(5):668.

12. Cao Y, Montgomery S, Ottosson J, Näslund E, Stenberg E. Deep Learning Neural Networks to Predict Serious Complications After Bariatric Surgery: Analysis of Scandinavian Obesity Surgery Registry Data. JMIR Med Inform. 2020 May 8;8(5):e15992.

13. Wise ES, Amateau SK, Ikramuddin S, Leslie DB. Prediction of thirty-day morbidity and mortality after laparoscopic sleeve gastrectomy: data from an artificial neural network. Surg Endosc. 2020 Aug;34(8):3590–3596.

14. Nudel J, Bishara AM, de Geus SWL, Patil P, Srinivasan J, Hess DT, et al. Development and validation of machine learning models to predict gastrointestinal leak and venous thromboembolism after weight loss surgery: an analysis of the MBSAQIP database. Surg Endosc. 2021 Jan;35(1):182–191.

15. Capretti G, Bonifacio C, De Palma C, Nebbia M, Giannitto C, Cancian P, et al. A machine learning risk model based on preoperative computed tomography scan to predict postoperative outcomes after pancreatoduodenectomy. Updates Surg. 2022 Feb;74(1):235–243.

16. Kambakamba P, Mannil M, Herrera PE, Müller PC, Kuemmerli C, Linecker M, et al. The potential of machine learning to predict postoperative pancreatic fistula based on preoperative, non-contrast-enhanced CT: A proof-of-principle study. Surgery. 2020 Feb;167(2):448–454.

17. Pfitzner B, Chromik J, Brabender R, Fischer E, Kromer A, Winter A, et al. Perioperative Risk Assessment in Pancreatic Surgery Using Machine Learning. Annu Int Conf IEEE Eng Med Biol Soc. 2021 Nov;2021:2211–2214.

18. Mu W, Liu C, Gao F, Qi Y, Lu H, Liu Z, et al. Prediction of clinically relevant Pancreatico-enteric Anastomotic Fistulas after Pancreatoduodenectomy using deep learning of Preoperative Computed Tomography. Theranostics. 2020;10(21):9779–9788.

19. Han IW, Cho K, Ryu Y, Shin SH, Heo JS, Choi DW, et al. Risk prediction platform for pancreatic fistula after pancreatoduodenectomy using artificial intelligence. World J Gastroenterol. 2020 Aug 14;26(30):4453–4464.

20. Shen Z, Chen H, Wang W, Xu W, Zhou Y, Weng Y, et al. Machine learning algorithms as early diagnostic tools for pancreatic fistula following pancreaticoduodenectomy and guide drain removal: A retrospective cohort study. Int J Surg. 2022 Jun;102:106638.

21. Yoon SJ, Kwon W, Lee OJ, Jung JH, Shin YC, Lim CS, et al. External validation of risk prediction platforms for pancreatic fistula after pancreatoduodenectomy using nomograms and artificial intelligence. Ann Surg Treat Res. 2022 Mar;102(3):147–152.

22. Zeng S, Li L, Hu Y, Luo L, Fang Y. Machine learning approaches for the prediction of postoperative complication risk in liver resection patients. BMC Med Inform Decis Mak. 2021 Dec 30;21(1):371.

23. Mai RY, Lu HZ, Bai T, Liang R, Lin Y, Ma L, et al. Artificial neural network model for preoperative prediction of severe liver failure after hemihepatectomy in patients with hepatocellular carcinoma. Surgery. 2020 Oct;168(4):643–652.

24. Wang J, Zheng T, Liao Y, Geng S, Li J, Zhang Z, et al. Machine learning prediction model for post-hepatectomy liver failure in hepatocellular carcinoma: A multicenter study. Frontiers in Oncology. 2022;12:2;12:986867.

25. Lei L, Wang Y, Xue Q, Tong J, Zhou CM, Yang JJ. A comparative study of machine learning algorithms for predicting acute kidney injury after liver cancer resection. PeerJ. 2020;8:e8583.

26. Dong JF, Xue Q, Chen T, Zhao YY, Fu H, Guo WY, et al. Machine learning approach to predict acute kidney injury after liver surgery. World Journal of Clinical Cases. 2021;9(36):11255.

27. Sofo L, Caprino P, Schena CA, Sacchetti F, Potenza AE, Ciociola A. New perspectives in the prediction of postoperative complications for high-risk ulcerative colitis patients: machine learning preliminary approach. Eur Rev Med Pharmacol Sci. 2020 Dec;24(24):12781–12787.

28. Mizuno S, Okabayashi K, Ikebata A, Matsui S, Seishima R, Shigeta K, et al. Prediction of pouchitis after ileal pouch-anal anastomosis in patients with ulcerative colitis using artificial intelligence and deep learning. Tech Coloproctol. 2022 Jun;26(6):471–478.

29. Hosaka H, Takeuchi M, Imoto T, Yagishita H, Yu A, Maeda Y, et al. Machine Learning-based Model for Predicting Postoperative Complications among Patients with Colonic Perforation: A Retrospective study. J Anus Rectum Colon. 2021;5(3):274–280.

30. Adams K, Papagrigoriadis S. Creation of an effective colorectal anastomotic leak early detection tool using an artificial neural network. Int J Colorectal Dis. 2014 Apr;29(4):437–443.

31. Azimi K, Honaker MD, Chalil Madathil S, Khasawneh MT. Post-Operative Infection Prediction and Risk Factor Analysis in Colorectal Surgery Using Data Mining Techniques: A Pilot Study. Surgical Infections. 2020 Nov 1;21(9):784–792.

32. Mazaki J, Katsumata K, Ohno Y, Udo R, Tago T, Kasahara K, et al. A Novel Predictive Model for Anastomotic Leakage in Colorectal Cancer Using Auto-artificial Intelligence. Anticancer Res. 2021 Nov;41(11):5821–5825.

33. Patrascu S, Cotofana-Graure GM, Surlin V, Mitroi G, Serbanescu MS, Geormaneanu C, et al. Preoperative Immunocite-Derived Ratios Predict Surgical Complications Better when Artificial Neural Networks Are Used for Analysis—A Pilot Comparative Study. JPM. 2023 Jan 1;13(1):101.

34. Soguero-Ruiz C, Hindberg K, Mora-Jiménez I, Rojo-Álvarez JL, Skrøvseth SO, Godtliebsen F, et al. Predicting colorectal surgical complications using heterogeneous clinical data and kernel methods. Journal of Biomedical Informatics. 2016 Jun 1;61:87–96.

35. Ohno Y, Mazaki J, Udo R, Tago T, Kasahara K, Enomoto M, et al. Preliminary Evaluation of a Novel Artificial Intelligence-based Prediction Model for Surgical Site Infection in Colon Cancer. Cancer Diagnosis & Prognosis. 2022;2(6):691.

36. Sohn S, Larson DW, Habermann EB, Naessens JM, Alabbad JY, Liu H. Detection of Clinically Important Colorectal Surgical Site Infection using Bayesian Network. J Surg Res. 2017 Mar;209:168–173.

37. Kocbek P, Fijacko N, Soguero-Ruiz C, Mikalsen KØ, Maver U, Povalej Brzan P, et al. Maximizing Interpretability and Cost-Effectiveness of Surgical Site Infection (SSI) Predictive Models Using Feature-Specific Regularized Logistic Regression on Preoperative Temporal Data. Computational and Mathematical Methods in Medicine. 2019 Feb 19;2019:1–13.

38. Dudurych I, Kelly ME, Aalbers AGJ, Abdul Aziz N, Abecasis N, Abraham-Nordling M, et al. Predicting outcomes of pelvic exenteration using machine learning. Colorectal Dis. 2020;22(12):1933–1940.

39. Shao S, Zhao Y, Lu Q, Liu L, Mu L, Qin J. Artificial intelligence assists surgeons’ decision-making of temporary ileostomy in patients with rectal cancer who have received anterior resection. Eur J Surg Oncol. 2022;Feb;49(2):433-439.

40. Grass F, Storlie CB, Mathis KL, Bergquist JR, Asai S, Boughey JC, et al. Challenges of Modeling Outcomes for Surgical Infections: A Word of Caution. Surgical Infections. 2021 Jun 1;22(5):523–531.

41. Ruan X, Fu S, Storlie CB, Mathis KL, Larson DW, Liu H. Real-time risk prediction of colorectal surgery-related post-surgical complications using GRU-D model. J Biomed Inform. 2022 Nov;135:104202.

42. Wen R, Zheng K, Zhang Q, Zhou L, Liu Q, Yu G, et al. Machine learning-based random forest predicts anastomotic leakage after anterior resection for rectal cancer. J Gastrointest Oncol. 2021;12(3):921–932.

43. Weller GB, Lovely J, Larson DW, Earnshaw BA, Huebner M. Leveraging electronic health records for predictive modeling of post-surgical complications. Stat Methods Med Res. 2018 Nov 1;27(11):3271–3285.

44. Chen D, Afzal N, Sohn S, Habermann EB, Naessens JM, Larson DW, et al. Postoperative bleeding risk prediction for patients undergoing colorectal surgery. Surgery. 2018 Dec;164(6):1209–1216.

45. Lin V, Tsouchnika A, Allakhverdiiev E, Rosen AW, Gögenur M, Clausen JSR, et al. Training prediction models for individual risk assessment of postoperative complications after surgery for colorectal cancer. Tech Coloproctol. 2022 Aug;26(8):665–675.

46. Chen KA, Stem J, Guillem JG, Gomez SM, Kapadia MR. Improved Prediction of Surgical Site Infection after Colorectal Surgery Using Machine Learning. Diseases of the Colon and Rectum. 2022;65(5):6.

47. Gelbard RB, Hensman H, Schobel S, Khatri V, Tracy BM, Dente CJ, et al. Random forest modeling can predict infectious complications following trauma laparotomy. J Trauma Acute Care Surg. 2019 Nov;87(5):1125–1132.

48. Eickhoff RM, Bulla A, Eickhoff SB, Heise D, Helmedag M, Kroh A, et al. Machine learning prediction model for postoperative outcome after perforated appendicitis. Langenbecks Arch Surg. 2022;407(2):789–795.

49. Xue Q, Wen D, Ji MH, Tong J, Yang JJ, Zhou CM. Developing Machine Learning Algorithms to Predict Pulmonary Complications After Emergency Gastrointestinal Surgery. Front Med. 2021;8:655686.

50. Soguero-Ruiz C, Fei WME, Jenssen R, Augestad KM, Álvarez JLR, Jiménez IM, et al. Data-driven Temporal Prediction of Surgical Site Infection. AMIA Annu Symp Proc. 2015;2015:1164–1173.

51. Deng H, Eftekhari Z, Carlin C, Veerapong J, Fournier KF, Johnston FM, et al. Development and Validation of an Explainable Machine Learning Model for Major Complications After Cytoreductive Surgery. JAMA Netw Open. 2022 May 2;5(5):e2212930.

52. Merath K, Hyer JM, Mehta R, Farooq A, Bagante F, Sahara K, et al. Use of Machine Learning for Prediction of Patient Risk of Postoperative Complications After Liver, Pancreatic, and Colorectal Surgery. J Gastrointest Surg. 2020 Aug;24(8):1843–1851.

53. Chen KA, Berginski ME, Desai CS, Guillem JG, Stem J, Gomez SM, et al. Differential Performance of Machine Learning Models in Prediction of Procedure-Specific Outcomes. J Gastrointest Surg. 2022 Aug;26(8):1732–1742.

54. Bunn C, Kulshrestha S, Boyda J, Balasubramanian N, Birch S, Karabayir I, et al. Application of machine learning to the prediction of postoperative sepsis after appendectomy. Surgery. 2021 Mar;169(3):671–677.

55. Hadaya J, Verma A, Sanaiha Y, Ramezani R, Qadir N, Benharash P. Machine learning-based modeling of acute respiratory failure following emergency general surgery operations. PLoS One. 2022 Apr 28;17(4):e0267733.

56. El Hechi MW, Maurer LR, Levine J, Zhuo D, El Moheb M, Velmahos GC, et al. Validation of the Artificial Intelligence-Based Predictive Optimal Trees in Emergency Surgery Risk (POTTER) Calculator in Emergency General Surgery and Emergency Laparotomy Patients. J Am Coll Surg. 2021 Jun;232(6):912-919.e1.

57. Bertsimas D, Dunn J, Velmahos GC, Kaafarani HMA. Surgical Risk Is Not Linear: Derivation and Validation of a Novel, User-friendly, and Machine-learning-based Predictive OpTimal Trees in Emergency Surgery Risk (POTTER) Calculator. Ann Surg. 2018 Oct;268(4):574–583.

58. Renganathan V. Overview of artificial neural network models in the biomedical domain. BLL. 2019;120(07):536–540.

59. Myles AJ, Feudale RN, Liu Y, Woody NA, Brown SD. An introduction to decision tree modeling. J Chemometrics. 2004 Jun;18(6):275–285.

60. Natekin A, Knoll A. Gradient boosting machines, a tutorial. Front Neurorobot. 2013 Dec 4;7:21.

61. Biau G, Scornet E. A random forest guided tour. TEST. 2016 Jun;25(2):197–227.

62. Noble WS. What is a support vector machine? Nat Biotechnol. 2006 Dec;24(12):1565–1567.

63. Kramer O. K-Nearest Neighbors. In: Dimensionality Reduction with Unsupervised Nearest Neighbors. Springer Berlin Heidelberg; 2013. p. 13–23.

64. Rish I. An empirical study of the naive Bayes classiﬁer. InIJCAI 2001 workshop on empirical methods in artificial intelligence 2001 Aug 4 (Vol 3, No 22, pp 41-46).
